# Antidiarrhoeal and antimicrobial activity of *Calpurnia aurea* leaf extract

**DOI:** 10.1186/1472-6882-13-21

**Published:** 2013-01-28

**Authors:** Shemsu Umer, Alemu Tekewe, Nigatu Kebede

**Affiliations:** 1 School of Pharmacy, Addis Ababa University, Addis Ababa, Ethiopia; 2 Akililu Lemma Institute of Pathobiology, Addis Ababa University, Addis Ababa, Ethiopia

**Keywords:** *Calpurnia aurea* leaf extract, Antidiarrhoeal, Antimicrobial, Ethiopia

## Abstract

**Background:**

In Ethiopia, *Calpurnia aurea* is used for the treatment of syphilis, malaria, rabies, diabetes, hypertension, diarrhoea, leishmaniasis, trachoma, elephantiasis, fungal diseases and different swellings. However, despite its traditional usage as an antidiarrhoeal and antimicrobial agent, there is limited or no information regarding its effectiveness and mode of action in diarrhoea which may be caused by *Shigella flexneri*, *Staphylococcus aureus*, *Escherichia coli* and *Salmonella typhi*. Hence, we evaluated the 80% methanol (MeOH) extract of dried and powdered leaves of *C. aurea* for its antidiarrhoeal and antimicrobial activities.

**Methods:**

Swiss albino mice of either sex were divided into five groups (five/group): Group I served as control and received vehicle (1% Tween 80) at a dose of 10 ml/kg orally; Group II served as standard and received loperamide at the dose of 3 mg/kg orally; Groups III, IV and V served as test groups and received the 80% MeOH leaf extract of *C. aurea* at doses of 100, 200 and 400 mg/kg orally, respectively. Diarrhoea was induced by oral administration of 0.5 ml castor oil to each mouse, 1 h after the above treatments. During an observation period of 4 h, time of onset of diarrhea, total number of faecal output (frequency of defecation) and weight of faeces excreted by the animals were recorded. Data were analyzed using one way analysis of variance followed by Tukey post test. Antimicrobial activity test was conducted using agar well diffusion assay. Clinical isolates tested were *Salmonella typhi, Salmonella paratyphi, Salmonella typhimurium, Shigella species, Pseudomonas aeruginosa, Staphylococcus aureus* and *Escherichia coli.*

**Results:**

In castor oil induced diarrhea model, the 80% methanol leaf extract of *C. aurea* at 100, 200 and 400 mg/kg and the standard drug loperamide (3 mg/kg) significantly reduced the time of onset of diarrhea, the frequency of defecation (total number of faecal output) and weight of faeces. *C. aurea* leaf extract also showed good antimicrobial activity against all tested organisms.

**Conclusions:**

*C. aurea* possesses good antidiarrhoeal and antimicrobial activity which support the traditional use of the plant in the treatment of diarrhea in Ethiopia.

## Background

Diarrhoeal disease is a leading cause of mortality and morbidity, especially among children in developing countries resulting in a major health care problem [1]. The major causative agents of diarrhoea in humans include: *Shigella flexneri*, *Staphylococcus aureus*, *Escherichia coli* and *Salmonella typhi*[2]. *Candida albicans* has also been known to cause diarrhoea in humans [3]. Despite the availability of vast spectrum of approaches for diarrhoeal management, vast majority of people in developing countries rely on herbal drugs for the management of diarrhoea. WHO has encouraged studies for treatment and prevention of diarrhoeal diseases depending on traditional medical practices [4].

Consumption of medicinal herbs is tremendously increasing over the past decade as alternative approach to improve the quality of life and maintain good health. Medicinal plants have been used for centuries as remedies for human diseases. Extensive studies of the adverse effects of these herbal medicines and establishment of a good correlation between biomarkers and plants are essential for ensuring the efficiency and quality of herbal medicines. Recently, there has been growing interest in exploiting biological activities of flora and fauna owing to their natural origin, cost effectiveness and lesser side effects. Plant-based natural constituents can be derived from any part of the plant like bark, leaves, flowers, roots, fruits, seeds, etc. Medicinal properties of plants unique to particular plant species or groups are consistent with the concept that combination of secondary products in a particular plant is taxonomically distinct [5].

The acceptance of traditional medicine as an alternative form of health care and the development of microbial resistance to the available antibiotics have led researchers to investigate the antimicrobial activity of herbal extracts. Plants containing flavonoids, terpenoids, steroids, phenolic compounds and alkaloids have been reported to have antimicrobial activity. WHO has continued a diarrhoeal disease control programme which includes studies of traditional medicinal practices together with the evaluation of health education and preventive approaches. This may reduce mortality rate in developing countries due to diarrhea [5].

In developing countries, majority of people almost exclusively use traditional medicines in treating all sorts of diseases, including diarrhoea. It would be interesting to search for plants with antidiarrhoeal and antimicrobial activities that could be used against any type of diarrhoeal disease. A range of medicinal plants with antidiarrhoeal and antimicrobial properties have been widely used by traditional healers. However, therapeutic potentials of some of these medicines have not been scientifically evaluated [1]. Among these plants, *Calpurnea aurea* which is widely distributed throughout tropical Africa enjoys a number of ethnomedical uses in Ethiopia. Traditionally, the leaves are used to cure diarrhoea, stomach-ache, bowel, and bladder disorders [6]. Therefore, it is necessary to establish the scientific basis for the therapeutic actions of traditional plant medicines as these may serve as the source for the development of more effective drugs.

The aim of the present study was to evaluate the possible antidiarrhoeal (*in vivo*) and antimicrobial (*in vitro*) properties of the leaf extract of *C. aurea*, in order to establish the claimed biological activities of this plant.

## Materials and methods

### Plant material

Leaves of *Calpurnea aurea* were collected from School of Pharmacy, Addis Ababa University, Addis Ababa, Ethiopia in October 2011. Identity of the plant was confirmed by a taxonomist at the National Herbarium, Addis Ababa University. The leaves were then dried at room temperature under shade and then ground to fine powder using sterile porcelain mortar and pistil.

### Preparation of the extract

One hundred grams of the dried and powdered plant was extracted with 80% methanol by maceration. The extract was filtered and concentrated using rotary vapor at a temperature of 40°C (Yield: 9.8% on dried weight).

### Preliminary phytochemical analysis

The methanol extract was tested for the presence or absence of alkaloids, flavonoids, tannins, terpenoids and saponins using the procedure described by Sofowara [7].

### Animals

Twenty five Swiss albino mice of either sex, weighing 20–30 g and aged 6–8 weeks were used for the experiment. The animals were obtained from animal center of Ethiopian Health and Nutrition Research Institute. They were kept in plastic cages at 22 ± 2°C and on a 12 h light–dark cycle with free access to pellet food and water. The animals were acclimatized to laboratory condition for one week prior to the experiments. All procedures performed were reviewed and approved by the Addis Ababa University, College of Health Sciences Review Board and conform to internationally accepted principles.

### Test strains

*Salmonella typhi, Salmonella paratyphi, Salmonella typhimurium, Shigella species, Pseudomonas aeruginosa, Staphylococcus aureus* and *Escherichia coli,* all clinical isolates were obtained from Akililu Lema Institute of Pathobiology, Microbiology Department, Addis Ababa University, Addis Ababa, Ethiopia.

### Acute oral toxicity test

In the acute oral toxicity study of methanol extract of *C. aurea*, a limit dose of each 2000 mg/kg body weight of the animal was administered on a single test animal orally by gavage. The limit test was repeated three times on a single test animal as a part of an oral acute toxicity assay. As no mortality of experimental animals was observed at the limit dose for the LD50 study, a dose regime of more than the limit dose, i.e., 5000 mg/kg body weight was planned and performed on a single test animal at a time and repeated three times [8].

### Castor oil induced diarrhea

The method described by Shoba and Thomas [9], was followed for this study with slight modification. The animals were all screened initially by giving 0.5 ml of castor oil one week before the actual experiment. Only those showing diarrhoea were selected for the final experiment. Twenty five mice fasted for 24 h were randomly allocated to five groups of five animals each. Group I (received 1% tween 80 at a dose of 10 ml/kg) served as control group, Group II received the standard drug loperamide 3 mg/kg, p.o. Group III, IV and V received the methanol leaf extract of *C. aurea* at the doses of 100, 200 and 400 mg/kg p.o., respectively. One hour after administration, all animals received 0.5 ml of castor oil and then they were individually place in cages the floor of which was lined with transparent paper. During an observation period of 4 h, the time of onset of diarrhoea, the total number of faecal output (frequency of defecation) and weight of faeces excreted by the animals were recorded.

### Antimicrobial activity

Antimicrobial activity was evaluated on the following intestinal pathogens: *Salmonella typhi, Salmonella paratyphi, Salmonella typhimurium, Shigella species, Pseudomonas aeruginosa, Staphylococcus aureus* and *Escherichia coli,* all are clinical isolates*.* Agar well diffusion method was used to determine antimicrobial activity. Diluted inoculums (0.1 mL) of test organism (10^6^ cfu/mL) were spread on Muller-Hinton agar plates. Wells of 8 mm diameter were punched into the agar medium with sterile cork borer under aseptic conditions and filled with 50 μl of 250 mg/ml of plant extract, solvent blank and standard antibiotic (gentamycin). The plate was kept at room temperature for 2 h for diffusion and was then incubated for 24 h at 37°C. Antimicrobial activity was evaluated by measuring the zone of inhibition against the test organisms [10]. Gentamycin (1 mg disc) was used as a reference standard and dimethylsulphoxide (DMSO) was used as a control. The growth was compared with the reference as well as the control. Each experiment was repeated three times.

Micro dilution broth method was used to determine minimum inhibitory concentration (MIC). 80% methanol leaf extract of *C. aurea* inhibiting growth of one or more microorganisms was tested for MIC. Serial dilutions were prepared from 250 mg/ml of the plant extract using DMSO to make 250, 125, 62.5, 31.25, and 15.625 mg/ml. The wells were inoculated with 0.1 mL aliquot of test organisms (10^6^ cfu/mL) having serial dilutions of the extract (50 μl, each). The micro plate was incubated at 37°C ± 1°C for 24 h. Dilution of the extract corresponding to respective test organism showing no visible growth was considered as MIC.

### Statistical analysis

The data are represented as mean ± SEM, and statistical significance was carried out employing one way analysis of variance (ANOVA) followed by Tukey post test where P < 0.05 was considered statistically significant.

## Results

### Phytochemical screening

Phytochemical screening of the 80% methanol extract of *C. aurea* leaf revealed the presence of alkaloids, tannins, flavonoids and saponins.

### Acute toxicity

The various observations showed normal behavior of the treated mice. No toxic effects were observed at a higher dose of 5 g/kg body weight. Hence, there were no lethal effects in any of the groups.

### Antidiarrhoeal effects

The 80% methanol extract of *Calpurnia aurea* leaves was found to be effective in a dose dependent manner against castor oil induced diarrhoea on experimental mice at all tested doses. At the dose of 400 mg/kg body weight, the extract produced a significant decrease in the severity of diarrhoea in terms of reduction in the rate of defecation and consistency of faeces in albino mice. At the same dose, the extract showed significant antidiarrhoeal activity (P < 0.001) showing 82.93% reduction in diarrhoea comparable to that of the standard drug loperamide that showed 87.80% reduction in diarrhoea (Table 1).

**Table 1:** **Effect of *Calpurnia aurea *extract on castor oil induced diarrhea in mice**

**Treatment**	**Dose, (mg/kg,p.o.)**	**Time of onset of diarrhea(min.)**	**Total number of faeces in 4 h(frequency of defecation in 4 h)**	**% Inhibition of defecation**	**Weight of stool(g)**
Group I		91 ± 10.9	8.2 ± 1.5	-	0.72 ± 0.07
Group II	3	237.8 ± 2.0^b^	1 ± 0.3^b^	87.80	0.04 ± 0.05^b^
Group III	100	145.2 ± 15.3^a^	4.8 ± 0.9^a^	41.46	0.30 ± 0.08^b^
Group IV	200	193 ± 14.5^b^	3.2 ± 0.2^c^	60.98	0.20 ± 0.05^b^
Group IV	400	212.4 ± 11.3^b^	1.4 ± 0.2^b^	82.93	0.01 ± 0.04^b^

Significantly different when compared with that of the control at ^a^P < 0.05, ^b^P < 0.001, ^c^P < 0.01, results are mean ± SEM.

### Antimicrobial activity

The 80% methanol extract of the leaves of C*. aurea* moderately inhibited the growth of all the tested bacterial strains. Gentamycin at a concentration of 1 mg/ml fully inhibited the growth of all the bacterial strains except *Shigella* spps (Table 2). Minimum inhibitory concentration (MIC) is the lowest concentration of an antimicrobial agent that will inhibit the visible growth of a microorganism after overnight incubation. The extract of *C. aurea* showed highest activity against *S.typhi* and *E. coli* among the tested microorganisms (Table 3).

**Table 2:** **Inhibition zones (mm) of 80% methanol extract of *Calpurnia aurea *leaves and Gentamycin**

**Organisms**	**Inhibition zones(mm)**	
	**Extract**	**Gentamycin**
*S. typhi*	10	18
*S. paratyphi*	10	16
*S. typhimurium*	9	16
*Shigella spps*	11	0
*P. aeuroginosa*	9	19
*S. aureus*	11	20
*E. coli*	14	20

**Table 3:** **Minimum inhibitory concentrations (MIC) of 80% methanol extract of *Calpurnia aurea *leaves**

**Organisms**	**Minimum inhibitory concentrations(mg/mL)**
*S. typhi*	31.25
*S. paratyphi*	125
*S. typhimurium*	62.5
*Shigella spps*	125
*P. aeuroginosa*	125
*S. aureus*	125
*E. coli*	31.25

## Discussion

People customarily using plant(s) or plant-derived preparations consider them to be efficacious against diarrheal disorders without any scientific basis to explain the action of such plants. The aim of this study was to experimentally evaluate the acclaimed use of *C. aurea* leaves, which are regarded to confer protection in diarrhoea in Ethiopian traditional medicine. Several studies have validated the use of antidiarrheal medicinal plants by investigating the biological activity of extracts of such plants, which have antispasmodic effects, delay intestinal transit, suppress gut motility, stimulate water adsorption, or reduce the intraluminal fluid accumulation [11]. This experimental model was therefore employed to validate antidiarrhoeal efficacy of *C. aurea* extract in the current study.

Diarrhoea may be characterized as the abnormally frequent defecation of faeces of low consistency which may be due to a disturbance in the transport of water and electrolytes in the intestines. Despite the multiplicity of aetiologies, the four major mechanisms responsible for the pathophysiology in water and electrolytes transport are (i) increased luminal osmolarity (osmotic diarrhoea), (ii) increased electrolytes secretion (secretory diarrhoea), (iii) decreased electrolytes absorption, and (iv) deranged intestinal motility causing a decreased transit time [12].

It is widely known that castor oil is metabolized into ricinoleic acid in the gut, which in turn irritates and causes inflammation in the intestinal mucosa, resulting in the release of inflammatory mediators, such as prostaglandins, histamine, and so forth. The prostaglandins thus released promote vasodilatation, smooth muscle contraction, and mucus secretion in the small intestines. Prostaglandins of the E series are considered to be good diarrheogenic agents in experimental animals as well as in human beings. The inhibitors of prostaglandins biosynthesis are therefore considered to delay castor oil–induced diarrhoea [13].

Methanol extract of *C. aurea* (100–400 mg/kg, p.o.) significantly (*p* < 0.05–0.001), reduced the faecal output produced by castor oil. At doses of 100–400 mg/kg (p.o.), the plant extract significantly (*p* < 0.05–0.001), and dose dependently delayed the onset of diarrhoea induced by castor oil when compared with the untreated controls. *C. aurea* (200 mg/kg, p.o.) reduced the number of fecal episodes by 60.98% while the dose of 400 mg/kg (p.o.) significantly (*p* < 0.001), reduced the number of animals suffering from diarrhoea by reducing defecation by 82.93%. Loperamide (3 mg/kg, p.o.) profoundly (*p* < 0.001), reduced the faecal output produced by castor oil. The onset of castor oil-induced diarrhoea and number of diarrhoeal episodes were also profoundly prolonged (*p* < 0.001), and reduced (*p* < 0.001), respectively by loperamide. Loperamide (3 mg/kg) reduced the number of fecal episodes by 87.80%. In terms of protection from diarrhea at 4 h, the 400 mg/kg dose of *C. aurea* extract was comparable with the standard drug loperamide (Table 1).

Anti-diarrhoea activity was found in plants possessing tannins, alkaloids, saponins, flavonoids, steroids and/or terpenoids [1,13,14]. Anti-diarrhoea activities of flavonoids have been ascribed to their ability to inhibit intestinal motility and hydroelectrolytic secretions which are known to be altered in diarrhoeic conditions [15]. Tannins present in anti-diarrhoea plants denature proteins in the intestinal mucosa by forming protein tannates which may reduce secretion. Studies on the functional role of tannins also reveal that they could also bring similar functions by reducing the intracellular Ca^2+^ inward current or by activation of the calcium pumping system (which induces the muscle relaxation) [16]. Phytochemical screening of *C. aurea* leaf extract revealed the presence of alkaloids, tannins, flavonoids and saponins. These constituents may be responsible for the *in vivo* anti-diarrhoea activity of *C. aurea*. At acute toxicity level, the extracts did not cause any mortality or visible signs of toxicity or differences in food and water uptake in the animals up to 5000 mg/kg.

The methanol extract of the leaves of *C. aurea* exhibited broad spectrum of antibacterial activity. It was observed in the present study that methanol extract inhibited the growth of all pathogenic bacteria tested moderately. Increased inhibition was found against *E. coli* and *Shigella* spp. MICs of this extract is summarized in Table 3. Preliminary phytochemical screening of *C. aurea* showed the presence of a number of bioactive constituents such as tannins, flavonoids, saponins, and alkaloids. The antimicrobial activity could be due to the presence of these phytoconstituents. Tannins and flavonoids in general have been reported to have antidiarrhoeal activity through inhibition of intestinal motility, antimicrobial action and antisecretory effects [17].

A study conducted in South Africa has shown that the antibacterial activity of methanol extracts of the leaves of *C. aurea* is much higher than that of the stem extract. It was also reported that *C. aurea* showed strong antibacterial activity comparable to that of standard gentamycin (0.1 mg/ml) though at MIC of 100 mg/ml. Moreover, the study also revealed that the plant extracts has *in vitro* broad spectrum antibacterial activity [18]. The result of our study also showed similar antimicrobial activity with better activity. These similarities indicate that the studied plant possess broad spectrum antimicrobial activities. However, there is a difference between our work and the previous reported data due to the environmental conditions under which the plant has grown as well as the microbes utilized.

## Conclusions

The present study validates the use of *Calpurnia aurea* leaves as anti-diarrhoeal agent in traditional medicine in Ethiopia.

## Competing interests

The authors declare that they have no competing interests.

## Authors’ contributions

SU: Collected the plant material, prepared the extract, carried out the antidiarrhoeal assays and drafted the manuscript. AT: Coordinated and conducted antimicrobial assay and helped in drafting manuscript. NK: Coordinated and conducted antimicrobial assay and provided all necessary materials for the assay. All authors read and approved the final manuscript.

## Pre-publication history

The pre-publication history for this paper can be accessed here:

http://www.biomedcentral.com/1472-6882/13/21/prepub
